# Endogenous arginase 2 as a potential biomarker for PEGylated arginase 1 treatment in xenograft models of squamous cell lung carcinoma

**DOI:** 10.1038/s41389-019-0128-0

**Published:** 2019-02-26

**Authors:** Sze-Kwan Lam, Sheng Yan, Shi Xu, Kin-Pong U, Paul Ning-Man Cheng, James Chung-Man Ho

**Affiliations:** 1 Division of Respiratory Medicine, Department of Medicine, The University of Hong Kong, Queen Mary Hospital, Hong Kong SAR, China; 2 School of Biomedical Sciences, The Chinese University of Hong Kong, Tai Po, Hong Kong SAR, China; 3 Bio-cancer Treatment International, 511-513, Bioinformatics Building, Hong Kong Science Park, Tai Po, Hong Kong SAR, China

## Abstract

Depletion of arginine induced by PEGylated arginase 1 (ARG1) (BCT-100) has shown anticancer effects in arginine auxotrophic cancers that lack argininosuccinate synthetase (ASS1) and ornithine transcarbamylase (OTC). High levels of endogenous arginase 2 (ARG2) have been previously reported in human lung cancers. Although a high-ARG2 level neither causes immunosuppression nor affects disease progression, it may theoretically affect the efficacy of PEGylated ARG1 treatment. ARG2 was shown to be highly expressed in H520 squamous cell lung carcinoma (lung SCC) xenografts but undetectable in SK-MES-1 and SW900 lung SCC xenografts. We propose that high-endogenous expression of ARG2 could impede the anti-tumor effect of PEGylated ARG1 in lung SCC. The in vivo effect of PEGylated ARG1 was investigated using three xenograft models of lung SCC. PEGylated ARG1 (60 mg/kg) suppressed tumor growth in SK-MES-1 and SW900 but not H520 xenografts. ASS1 was expressed in SK-MES-1 and SW900 xenografts while OTC expression remained low in all xenografts. A high-endogenous ARG2 level was detected only in H520 xenografts. Serum arginine level was decreased significantly by PEGylated ARG1 in all xenografts. Nonetheless intratumoral arginine level was decreased by PEGylated ARG1 in SK-MES-1 and SW900, not H520 xenografts. In SK-MES-1 xenografts, PEGylated ARG1 treatment induced G1 arrest, downregulation of Ki67 and Mcl-1 and activation of apoptosis. In SW900 xenografts, upregulation of Bim and activation of apoptosis were observed upon PEGylated ARG1 treatment. Silencing of ARG2 re-sensitized the H520 xenografts to PEGylated ARG1 treatment, partially mediated through arginine depletion via G1 arrest and apoptosis. PEGylated ARG1 treatment (BCT-100) was effective in lung SCC xenografts with low-endogenous levels of ASS1/OTC and ARG2. High-endogenous ARG2 expression may cause resistance to PEGylated ARG1 treatment in lung SCC xenografts. ARG2 may serve as a third predictive biomarker in PEGylated ARG1 treatment in lung SCC.

## Introduction

According to official statistics, lung cancer was the third most common cancer and the leading cause of cancer death in Hong Kong in 2015 (Hong Kong Cancer Registry 2015, http://www3.ha.org.hk/cancereg/default.asp) and worldwide in 2012 (Globocan 2012, http://globocan.iarc.fr/Default.aspx). Lung cancer can be divided into non-small-cell lung carcinoma and small-cell lung carcinoma. Non-small-cell lung carcinoma can be sub-divided into adenocarcinoma, squamous cell carcinoma (lung SCC) and large cell carcinoma. Cytotoxic chemotherapy (e.g., cisplatin/gemcitabine) and immunotherapy (e.g., nivolumab) are the major therapeutic approaches for lung SCC^1^. Novel approaches are urgently needed.

Arginine can be interconverted in the urea cycle (arginine→ornithine→citrulline→argininosuccinate→arginine, by arginase, ornithine transcarbamylase (OTC), argininosuccinate synthetase (ASS1), and argininosuccinate lyase, respectively). Normal cells have intact urea cycle enzymes so arginine is a nonessential amino acid. Tumor cells with an incomplete urea cycle due to an intrinsic deficiency in key enzymes will suffer arginine depletion. As such, arginine-degrading enzymes (arginase and arginine deiminase) have been investigated in the treatment of cancers that are sensitive to arginine depletion^2,3^. Co-expression of ASS1 and OTC in tumors is a well-known negative predictive biomarker for arginase treatment^4^.

BCT-100 is a PEGylated arginase 1 (ARG1) that is manufactured by Bio-Cancer Treatment International Limited in Hong Kong (US FDA IND granted in March 2012). It has shown anticancer activity in hepatocellular carcinoma (HCC)^2,5^ acute myeloid leukemia^6^, melanoma^7^, and mesothelioma^8^ in vitro and/or in vivo via induction of apoptosis or concurrently with cell cycle arrest. A phase I/II clinical trial of BCT-100 for treatment of HCC has shown it to be well tolerated with consequent increased progression-free survival for patients with adequate serum arginine depletion to less than 8 μM^4^. Interestingly, it has been reported that arginase 2 (ARG2) was highly expressed in some human lung cancers and neither affected disease progression nor suppressed the immune system^9^. High-endogenous ARG2 in tumor cells may lower the intratumoral arginine level similar to ARG1 treatment with cells adapting to a low intratumoral arginine environment. As such, high-endogenous ARG2 may theoretically depress the efficacy of PEGylated ARG1 treatment. Our preliminary result revealed that ARG2 expression was present in H520, but not SK-MES-1 or SW900 lung SCC xenografts. We hypothesized that a high-basal ARG2 level affected the anticancer effect of PEGylated ARG1 in lung SCC. ARG2 may serve as a third predictive biomarker in PEGylated ARG1 treatment.

## Results

### Endogenous ASS1 and ARG2 expression as well as tumor xenograft growth suppression with BCT-100

**Fig. 1:**
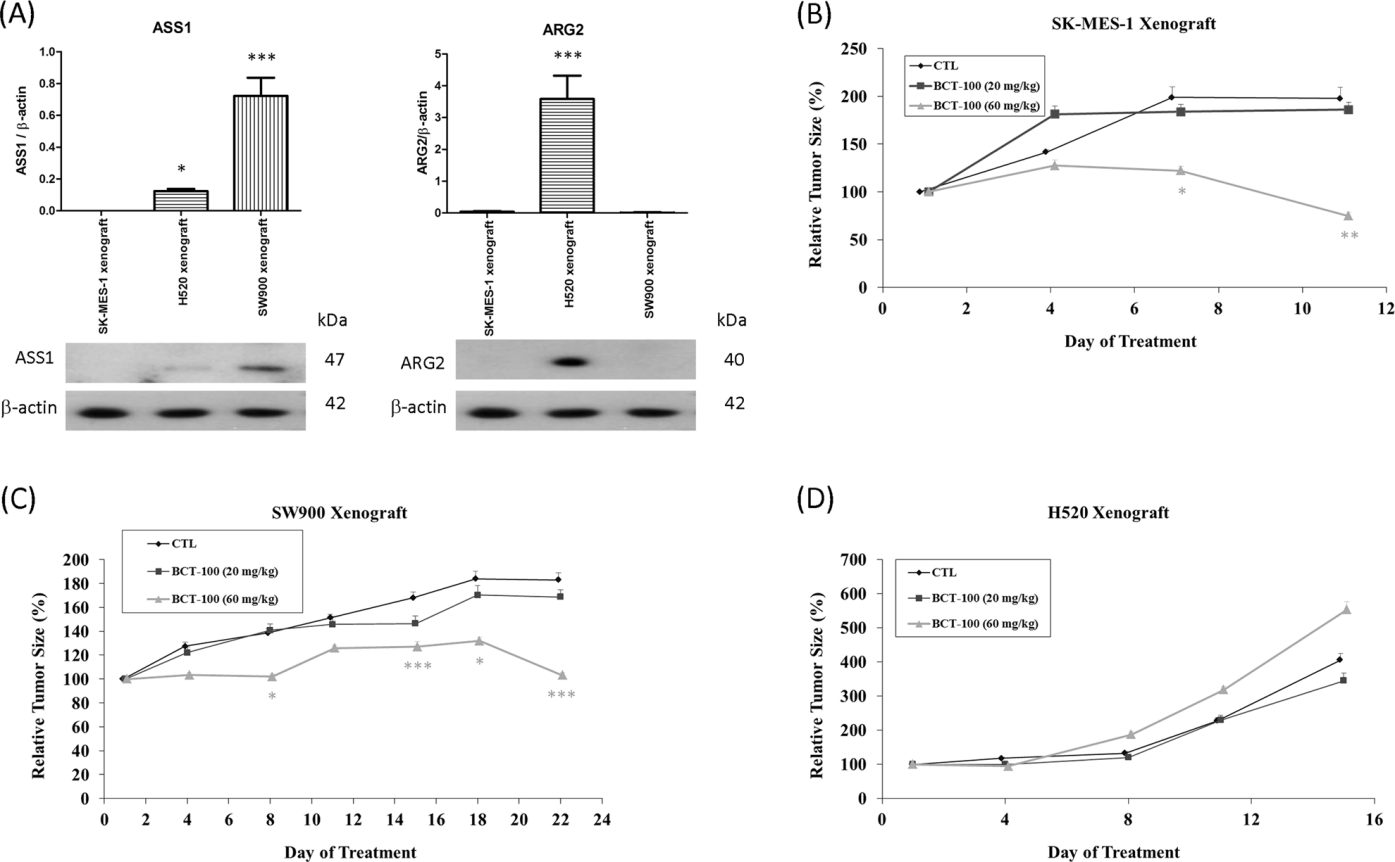
Endogenous ASS1 and ARG2 level as well as tumor suppression effect of BCT-100 in lung SCC xenograft models. **a** Endogenous ASS1 was found in H520 and SW900 xenografts while ARG2 was highly expressed in H520 xenografts. Protein expression levels of H520 and SW900 were compared with SK-MES-1 xenograft. Data represent the mean ± standard error of the mean (SEM) (*N* = 3) was analyzed using one-way analysis of variance (ANOVA) by Prism. BCT-100 inhibited tumor growth in **b** SK-MES-1 and **c** SW900, but not **d** H520 xenografts. Data represent the mean ± SEM (*N* = 6), and was analyzed using repeated measure ANOVA by Prism. A *p* value < 0.05 defined statistical significance (^*^p < 0.05, ^**^p < 0.01, ^***^p < 0.001)

The endogenous ASS1 level was undetectable, mildly expressed and highly expressed in SK-MES-1, H520 and SW900 xenografts, respectively. Basal ARG2 was highly expressed in H520 xenografts only (Fig. 1a). All xenografts were ARG1 and OTC negative (data not shown). BCT-100 treatment was started when tumors were clearly established after inoculation of SK-MES-1, H520 or SW900 cells. BCT-100 (60 mg/kg) suppressed tumor growth in SK-MES-1 (Fig. 1b) and SW900 (Fig. 1c), but not H520 xenografts (Fig. 1d). The growth rates of the three xenografts were different, resulting in variable duration of experiments. There was no significant difference in body weight among different groups during treatment (data not shown).

### Intratumoral BCT-100 level as well as serum and intratumoral arginine levels in xenografts

**Fig. 2:**
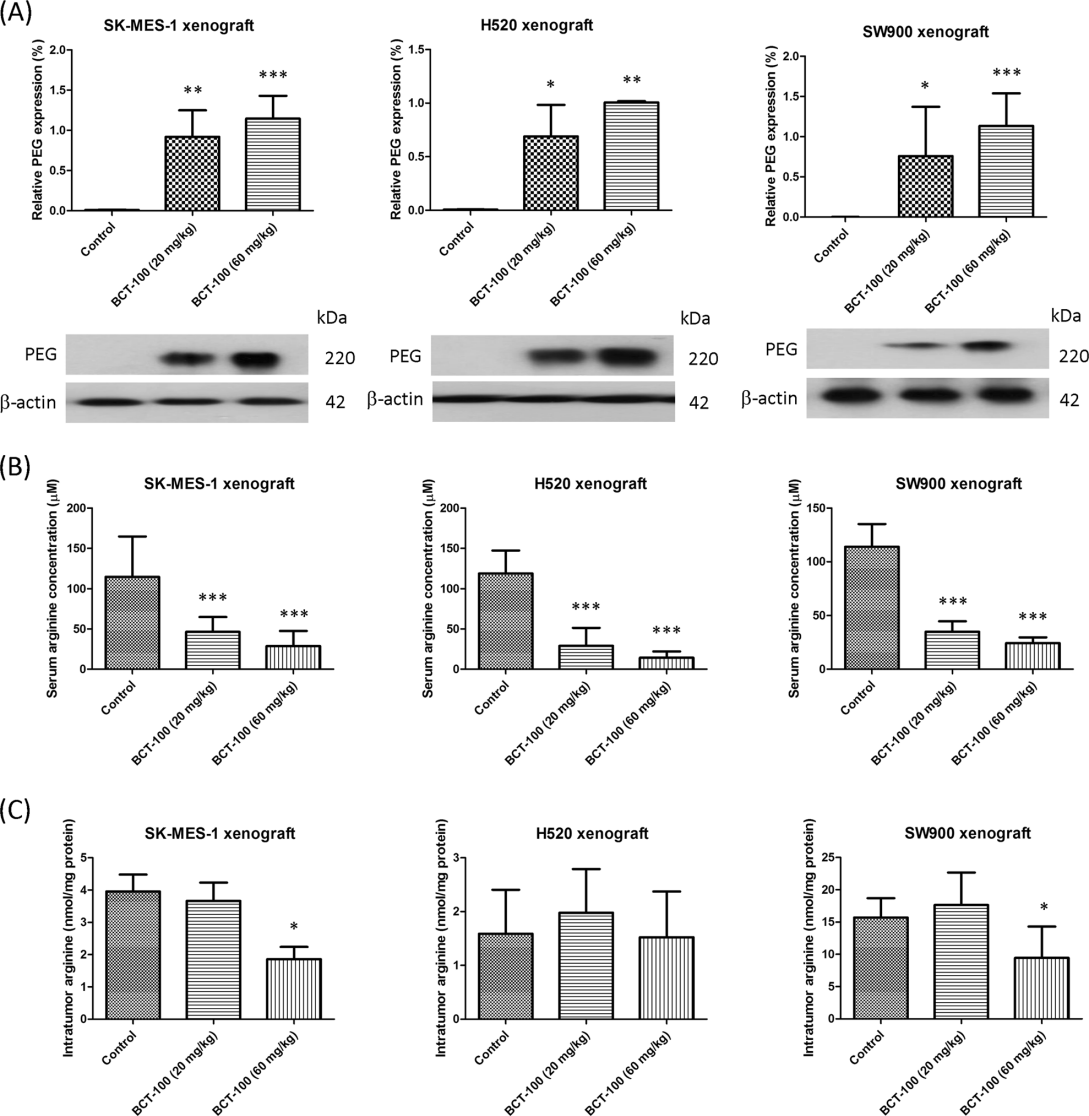
Accumulation of intratumoral BCT-100 as well as serum and intratumoral arginine depletion in xenograft models. **a** Intratumoral level of PEG-BCT-100 was increased in BCT-100 treatment arms in all xenografts. **b** BCT-100 decreased serum arginine concentration in all xenografts. **c** BCT-100 (60 mg/kg) reduced intratumoral arginine level in SK-MES-1 and SW900, but not H520 xenografts. Data represent the mean ± SEM (*N* = 6). A *p* value < 0.05 defined statistical significance (^*^p < 0.05, ^**^p < 0.01, ^***^p < 0.001)

Anti-PEG antibody can be used to detect the relative amount of BCT-100 in samples^8^. Intratumoral BCT-100 was found in all BCT-100 treatment arms in all xenograft models (Fig. 2a).

The serum arginine concentration in the control group was similar in all xenograft models with an average concentration of 116.1 ± 14.7 μM (mean ± SEM). In SK-MES-1 xenografts, serum arginine concentration decreased to 46.4 ± 7.6 and 28.7 ± 7.7 μM in 20 and 60 mg/kg BCT-100 treatment groups, respectively (*p* < 0.001). In H520 xenografts, the serum arginine concentration reduced to 29.2 ± 9.1 and 14.3 ± 3.2 μM in 20 and 60 mg/kg BCT-100 treatment groups, respectively (*p* < 0.001). Similarly, in SW900 xenografts, the serum arginine concentration declined to 34.9 ± 4.0 and 24.2 ± 2.2 μM in 20 and 60 mg/kg BCT-100 treatment groups, respectively (*p* < 0.001) (Fig. 2b).

In SK-MES-1 xenografts, intratumoral arginine content decreased from 4.0 ± 0.6 nmole/mg protein (mean ± SEM) in the control arm to 1.9 ± 0.5 nmole/mg protein (60 mg/kg BCT-100 arm, *p* < 0.05). In H520 xenografts, intratumoral arginine content remained unchanged in all arms. In SW900 xenografts, intratumoral arginine content declined from 15.7 ± 3.0 nmole/mg protein in the control group to 9.5 ± 4.8 nmole/mg protein in the 60 mg/kg BCT-100 arm, *p* < 0.05 (Fig. 2c).

### G1 arrest induced by BCT-100 in SK-MES-1 xenografts

**Fig. 3:**
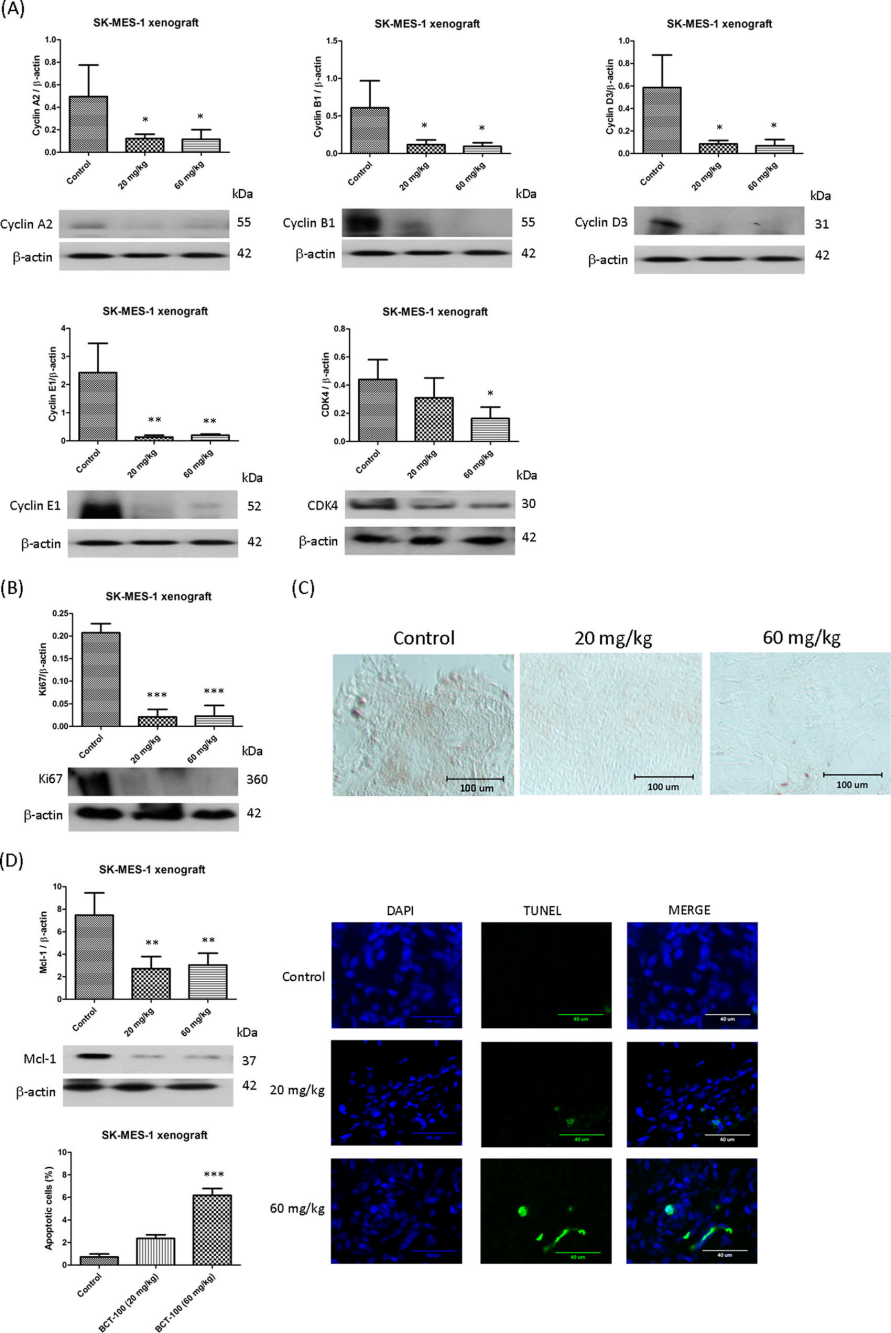

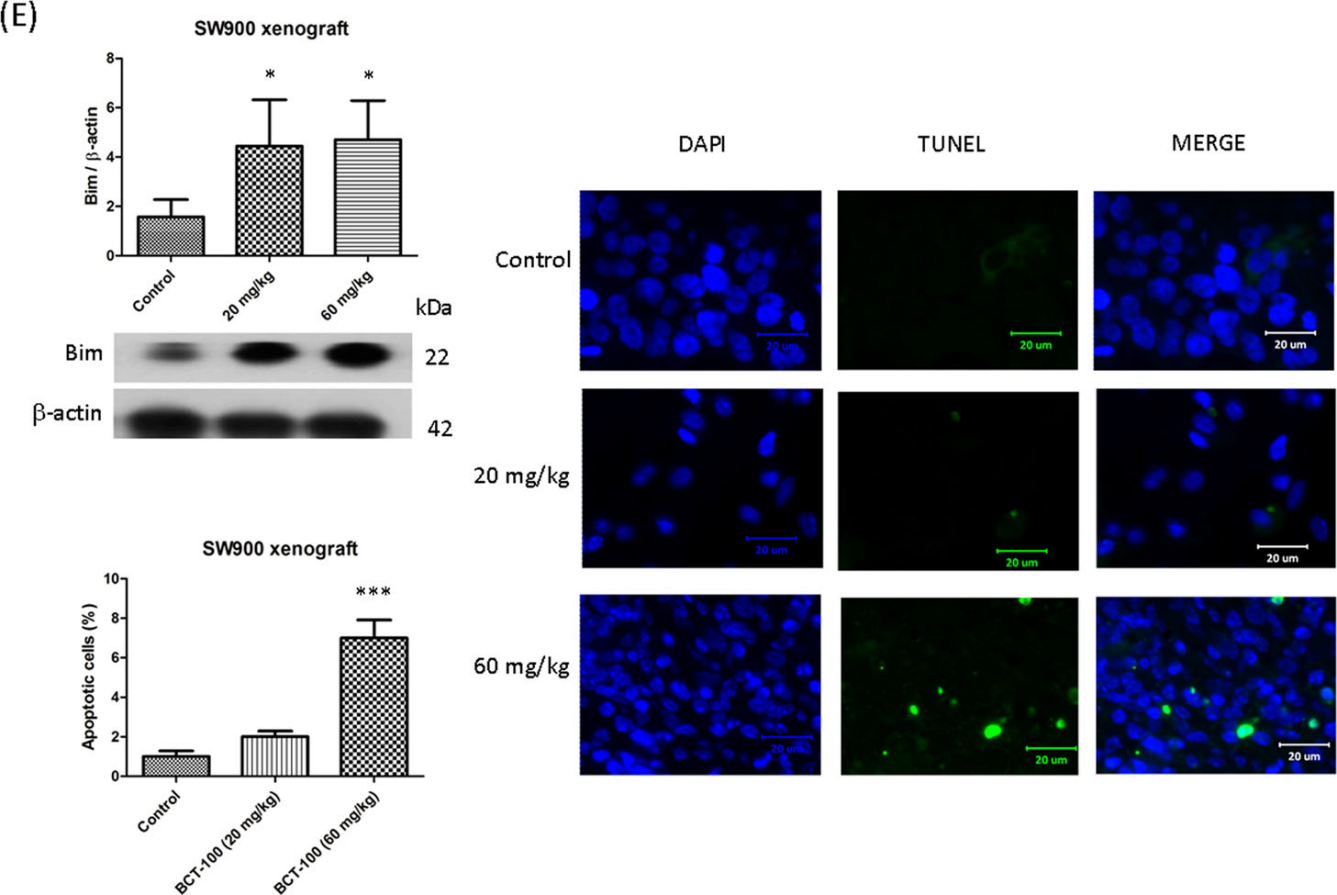
BCT-100 induced apoptosis or concurrently with G1 arrest in SK-MES-1 and SW900 xenografts. **a** BCT-100 reduced the expression of cyclin A2, B1, D3, E1, and CDK4 in SK-MES-1 xenografts. Expression of antiproliferation factor Ki67 was decreased as shown in **b** Western blots and **c** IHC in SK-MES-1 xenografts. **d** In SK-MES-1 xenografts, downregulation of Mcl-1 as well as increase in green TUNEL signal in BCT-100 treatment arms. **e** In SW900 xenografts, BCT-100 induced upregulation of Bim and increased green TUNEL signal. Data represent the mean ± SEM (*N* = 6). A *p* value < 0.05 defined statistical significance (^*^p < 0.05, ^**^p < 0.01, ^***^p < 0.001)

G1 arrest was indirectly evidenced by altered expression of related proteins in the cell cycle. Cyclin A2, B1, D3, E1, and CDK4 were downregulated by BCT-100 in SK-MES-1 xenografts (Fig. 3a). In addition, Ki67 expression was lower in treatment arms as demonstrated by both Western blot (Fig. 3b) and immunohistochemistry (IHC) staining (Fig. 3c). However, no alterations were observed in SW900 xenografts (data not shown).

### Apoptosis induced by BCT-100 in SK-MES-1 and SW900 xenograft models

Mcl-1 is an anti-apoptotic protein. Mcl-1 was downregulated in both 20 and 60 mg/kg BCT-100 treatment arms in SK-MES-1 xenografts (Fig. 3d). Mcl-1 expression was not changed in SW900 xenografts (data not shown). Bim is a pro-apoptotic protein. Upregulation of Bim was observed in the 60 mg/kg BCT-100 treatment group in SW900 xenografts (Fig. 3e). The expression of Bim was unaltered in SK-MES-1 xenografts (data not shown). In TUNEL assay, TUNEL-positive DNA strand breaks were noted in the BCT treatment arm (60 mg/kg) in both SK-MES-1 (Fig. 3d) and SW900 (Fig. 3e) xenografts. Both Mcl-1 and Bim belong to BCL-2 protein family which is responsible for early phase apoptosis. Although low dose of BCT-100 (20 mg/kg) could alter expression of Mcl-1 or Bim, it failed to induce DNA fragmentation (late phase apoptosis) as demonstrated in TUNEL assay.

### Silencing of ARG2 in H520 xenografts

**Fig. 4:**
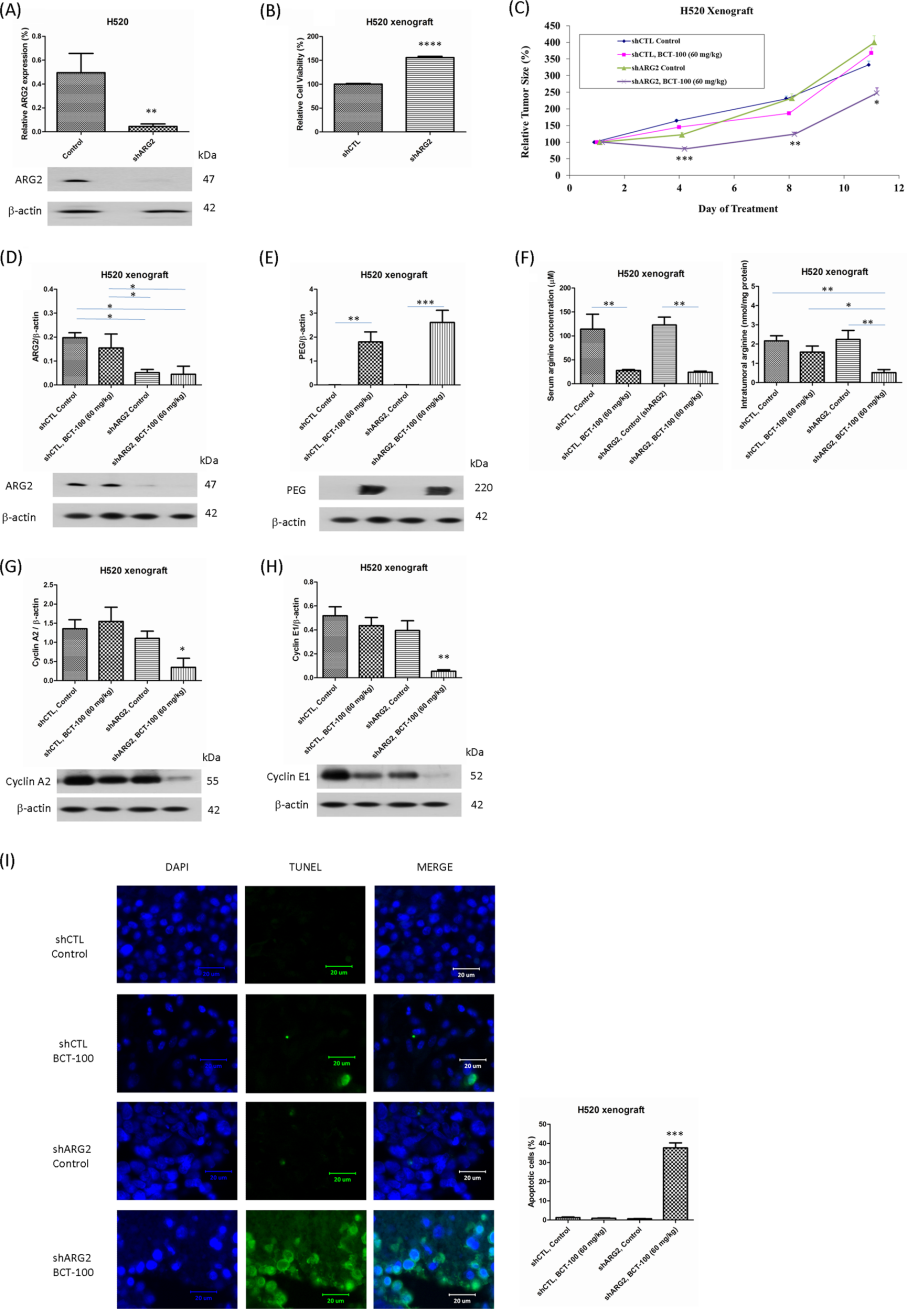
Silencing of ARG2 resensitized H520 xenografts to BCT-100 treatment via arginine depletion, G1 cell cycle arrest and apoptosis. **a** Expression of ARG2 was decreased after transduction of shARG2 in in vitro. **b** Cell viability was increased by about 50% after transduction of shARG2. **c** Silencing of ARG2 re-sensitized the H520 xenografts to BCT-100 treatment. Data were analyzed using repeated measure ANOVA by Prism. **d** ARG2 expression remained low after silencing of ARG2 in vivo. **e** Intratumoral PEG-BCT-100 was increased after BCT-100 treatment with or without silencing of ARG2. **f** Serum arginine level declined after BCT-100 treatment with or without silencing of ARG2. Intratumoral arginine level decreased in the BCT-100 treatment arm after silencing of ARG2. Downregulation of **g** cyclin A2 and **h** E1 as well as **i** increase in TUNEL signal were observed in the BCT-100 treatment group after silencing of ARG2. Data represent the mean ± SEM (*N* = 6). A *p* value < 0.05 defined statistical significance (^*^p < 0.05, ^**^p < 0.01, ^***^p < 0.001)

Expression of ARG2 was significantly decreased after transduction of shRNA targeting ARG2 (shARG2) (Fig. 4a). Cell viability was increased by about 50% after shARG2 treatment (Fig. 4b). In the absence of BCT-100 treatment, tumor volume was comparable in the control shRNA (shCTL) and shARG2 arms. Interestingly, although the shCTL group remained resistant to BCT-100, silencing of ARG2 resensitized H520 xenografts to BCT-100 treatment (Fig. 4c). Endogenous ARG2 remained low in the shARG2 and shARG2/BCT-100 arms when compared with shCTL and shCTL/BCT-100 groups (Fig. 4d). The expression of ASS1 and OTC remained unchanged after knockdown of ARG2 (data not shown). BCT-100 penetrated the tumor as reflected by a high-PEG level in the shCTL/BCT-100 and shARG2/BCT-100 treatment groups (Fig. 4e). Serum arginine level declined in the shCTL/BCT-100 and shARG2/BCT-100 arms (Fig. 4f) although intratumoral arginine level decreased only in the shARG2/BCT-100, not the shCTL/BCT-100 group (Fig. 4f). At the same time, cyclins A2 (Fig. 4g) and E1 (Fig. 4h) were downregulated and the TUNEL signal (apoptosis) was greatly increased in the shARG2/BCT-100 arm (Fig. 4i). Expression of Mcl-1 and Bim were unaltered in different treatment groups (data not shown).

### ASS1, OTC, and ARG2 expression in lung SCC patient samples

High-endogenous ARG2 expression has been reported in some lung SCC patients^9^ although the basal expressions of ASS1 and OTC are still unknown. We aimed to examine the endogenous ASS1, OTC, and ARG2 level in 12 lung SCC patient samples that may provide insights on potential clinical use of PEGylated ARG1.

**Fig. 5:**
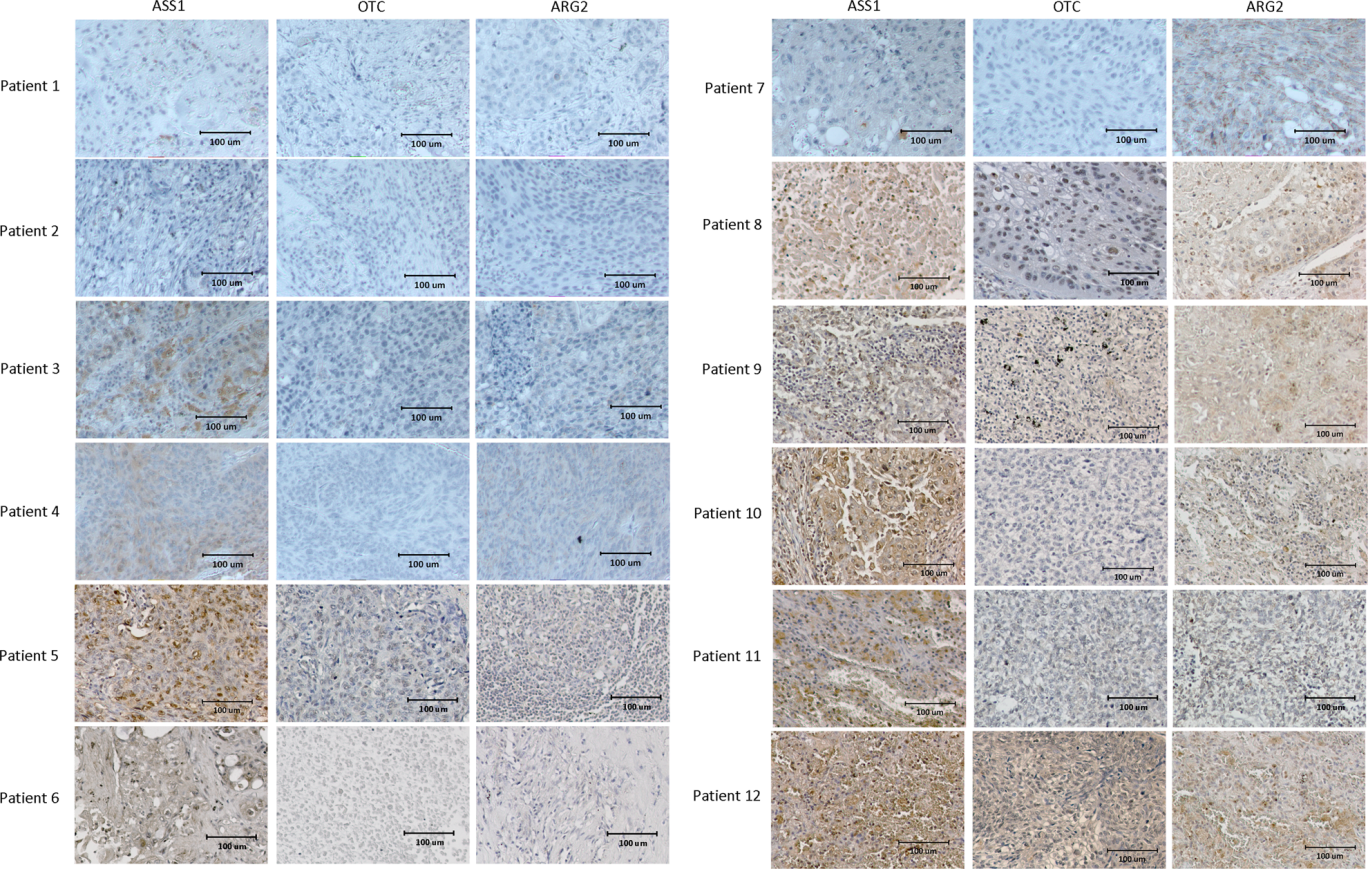
Basal expression of ASS1, OTC, and ARG2 in tumor samples of lung SCC patients. Target proteins (ASS1, OTC, and ARG2) were stained for 1 min with DAB (3,3′-diaminobenzidine). The nucleis were counterstained for 5 s with hematoxylin. ASS1 was highly expressed in ten tumors tumors. OTC expression was detectable in only one tumor. ARG2 was highly expressed in six tumors

The clinical characteristics of included patients as well as expression of ASS1, OTC, and ARG2 are summarized in Table 1. ASS1 was highly expressed in ten patients. OTC expression was positive in one patient only. Expression of ARG2 was high in six patients (Fig. 5).

**Table 1:** The clinical characteristics of included patients as well as expression of ASS1, OTC, and ARG2

Patient	Age	Sex	Smoking history	Differentiation	Stage	ASS1	OTC	ARG2
1	82	M	EX	PD	II	−	−	−
2	65	M	EX	PD	II	−	−	−
3	67	M	EX	MD	II	+	−	−
4	75	M	EX	PD	I	+	−	−
5	56	M	EX	PD	I	+	−	−
6	60	M	SM	MD	III	+	−	−
7	73	M	EX	PD	II	+	−	+
8	75	M	SM	MD	I	+	−	+
9	78	M	SM	PD	II	+	−	+
10	55	M	SM	PD	III	+	−	+
11	64	M	SM	MD	III	+	−	+
12	67	M	EX	MD	I	+	+	+

*EX* ex-smoker, *SM* current smoker, *MD* moderately differentiated carcinoma, *PD* poorly differentiated carcinoma

## Discussion

In SK-MES-1 and SW900 xenograft models, PEGylated ARG1 (BCT-100, 60 mg/kg) downregulated the intratumoral arginine level and suppressed tumor growth via apoptosis or concurrently with G1 arrest. Although the H520 xenograft model was OTC negative, it was resistant to PEGylated ARG1 treatment, possibly due to a high-endogenous ARG2 level. Silencing of ARG2 in the H520 xenografts resensitized this model to PEGylated ARG1 treatment. Endogenous ARG2 may serve as a third potential predictive biomarker in PEGylated ARG1 treatment of lung SCC.

There are two subtypes of arginases: ARG1 and ARG2. They have 100% homology at the active site and thus share the same biological function: conversion of arginine to ornithine. The tissue specificity of ARG1 is mainly to liver and red blood cells, and for ARG2, the kidney, brain, gastrointestinal tract, and prostate. The subcellular location of ARG1 and ARG2 is cytosol and mitochondria, respectively. ARG1 and ARG2 can be inhibited by proline and isoleucine, respectively^10^.

We have previously demonstrated that intratumoral arginine depletion was more important than systemic arginine depletion in BCT-100-treated mesothelioma xenograft models^8^. This phenomenon is particularly relevant in this study. In the H520 xenografts, PEGylated ARG1 or BCT-100 treatment was unable to alter the intratumoral arginine level. Since the biological functions of ARG1 and ARG2 are the same, a high-endogenous ARG2 level in these xenografts might also decrease the intratumoral arginine concentration. As such, the tumor cells may adapt to a low-arginine condition and be unaffected by PEGylated ARG1 treatment. After silencing of ARG2 with shARG2, basal intratumoral arginine level was not increased in the shARG2 control arm. The extra arginine might have been utilized in supporting cell growth as evidenced by increased cell viability after shARG2 treatment. Silencing of ARG2 increased cell viability and indicated that ARG2 might be a tumor suppressor in vitro. Expression of tumor suppressor in the tumor is not uncommon, the best well-known examples are PTEN^11^ and p53^12^. Indeed, ARG2 has been shown to act as a tumor suppressor in renal cell carcinoma^13^ which is concordant with this study. Nonetheless the tumor in the shARG2 arm grew no faster than that in the shCTL group in vivo. This is in line with the clinical observation that ARG2 expression does not affect disease progression^9^. We cannot explain the discrepancy between our in vitro and in vivo result in cell proliferation/tumor growth following shARG2 knockdown. It may be that the expression of genes related to cell survival, differentiation, proliferation, and resistance to therapy are different to those in 2D cultures (in vitro) and tumor spheroids (ex vivo)/xenograft models (in vivo)^14^. At the same time, the H520 xenografts were sensitive to PEGylated ARG1 (BCT-100) treatment after ARG2 knockdown. Since the biological functions of ARG1 and ARG2 are identical, silencing of ARG2 (tumor suppressor) was replenished by the excessive amount of PEGylated ARG1 (BCT-100) that caused arginine depletion and suppression of tumor growth. The mechanisms involved were cell cycle arrest and apoptosis, similar to other BCT-100-treated xenograft models in this study.

In addition, the basal expression of ASS1, OTC, and ARG2 in 12 human lung SCC clinical samples was examined. One sample was ASS1^+^OTC^+^ARG2^+^, hence resistance to PEGylated ARG1 treatment would be anticipated. Among the remaining 11 cases, 2 tumor samples were ASS1^−^OTC^−^ and 9 were ASS1^+^OTC^−^, all would be expected responders due to their low-endogenous ASS1/OTC expression. However, examination of ARG2 expression further identified five out of the nine tumors with ASS1^+^OTC^−^ARG2^+^ that would implicate possible resistance to PEGylated ARG1 treatment. It has been shown that cigarette smoke induced vascular stiffness and endothelial vascular dysfunction via activation of ARG2 using an ARG2 knockout C57BL/6J mouse model^15^. In this small series, 80% of current smokers had higher ARG2^+^ when compared to merely 28.6% among ex-smokers. As such, current smoking patients might potentially have less benefit from PEGylated ARG1 treatment due to higher endogenous ARG2 level. Despite the relatively small sample size, our findings in this study may provide insight in delineating predictive biomarkers in PEGylated ARG1 treatment using endogenous expression of ARG2. The clinical significance of our finding will need to be validated in a future clinical trial of PEGylated ARG1 in lung SCC.

Cationic amino acid transporter (CAT) has been shown to control arginine uptake and drug resistance in PEGylated ARG1 treatment in acute myeloid leukemia^16^. The expression of CAT-1 was undetectable in different treatment arms of all xenografts (data no shown) which indicated that CAT might have limited role in lung SCC models.

PEGylated ARG1 has been shown to induce apoptosis or concurrently with cell cycle arrest in human hepatoma^2^, HCC^17^, melanoma^7^, acute myeloid leukemia cells^16^, colorectal cancer^18^, small-cell lung carcinoma^19^, some of lung adenocarcinoma^20^, and mesothelioma^8^. Similar results were observed in this study. Key markers in the Akt/ERK pathway as well as reactive oxygen or nitrogen species were unaltered (data not shown) after arginine depletion by PEGylated ARG1 treatment in all lung SCC xenograft models. It seems that apoptosis and cell cycle arrest are the major mechanisms by which cancer cell growth is suppressed by PEGylated ARG1 treatment.

A phase I/II clinical trial of PEGylated ARG1 (BCT-100) in the treatment of advanced HCC was reported in 2015. Progression-free survival was improved in patients with adequate arginine depletion (<8 μM serum arginine concentration)^4^. Change in intratumoral arginine level was not investigated^4^ although it might play a more important role than serum arginine concentration as demonstrated in lung SCC in this and our previous studies^8^. We propose that depletion of intratumoral, on top of serum, arginine level may serve as a better biomarker for tumor response, which needs to be elucidated in future clinical trials. Moreover, the basal expression of ASS1 and OTC (well-known predictive biomarkers in PEGylated ARG1 treatment) were not checked in this clinical trial but should be incorporated in another phase II clinical study^4^. More importantly, endogenous ARG1 expression in HCC patients has been reported from seven studies: on average, 92% and 100% of all HCC and well-differentiated HCC patients, respectively, were ARG1 positive^21^. Since a high-endogenous ARG2 level impedes the efficacy of PEGylated ARG1 treatment in lung SCC xenografts, the same may also apply to endogenous ARG1 in HCC. The relationship of endogenous ARG1 with efficacy of PEGylated ARG1 treatment in HCC patients remains to be elucidated.

In conclusion, BCT-100 demonstrated significant in vivo anti-tumor effects in lung SCC, mediated by intratumoral arginine depletion with consequent apoptosis or concurrently with G1 arrest. ARG2 may serve as a potential predictive biomarker, in addition to ASS1 and OTC, for PEGylated ARG1 treatment in lung SCC.

## Materials and methods

### Cell lines and reagents

A panel of three squamous cell lung carcinoma cell lines (SK-MES-1, H520, and SW900) was obtained (American Type Culture Collection, Manassas, VA, USA) and incubated in MEM, RPMI-1640 and L-15 medium respectively (Gibco^®^, Life Technologies, Carlsbad, CA, USA) supplemented with 10% fetal bovine serum (Gibco^®^) in a humidified atmosphere of 5% CO_2_ at 37 °C. Cell lines were authenticated by ATCC in Dec 2016 by comparing the ATCC reference database profile and used within ten passages. No mycoplasma contamination was tested.

### PEGylated arginase 1 (BCT-100)

PEGylated arginase 1 (BCT-100, PEG-BCT-100 or rhArg1peg5000) was provided free-of-charge by Bio-cancer Treatment International Limited.

### Short hairpin RNA (shRNA) lentiviral particles transduction

Silencing of ARG2 in H520 cells was carried out according to the manufacturer’s protocol. All reagents were purchased from Santa Cruz. Briefly, cells were transduced overnight in complete medium containing polybrene (5 μg/ml), lentiviral particles and control shRNA (shCTL) (SC-108080) or shRNA targeting ARG2 (shARG2) (SC-29729-V). Stable clones were selected by addition of puromycin (5 μg/ml).

### Cell viability assay

A standard 3-(4,5-dimethylthiazol-2-yl)-2,5-diphenyltetrazolium bromide (MTT) cell viability assay was performed to test the effect of ARG2 silencing on cell viability^22^. Briefly, after shRNA transduction cells were stained with MTT for 2 h, followed by addition of DMSO after removal of all medium. Cells transduced with shCTL served as control (100% viability) while cells transduced with shARG2 were considered the treatment arm. Absorbance (595 nm) was measured using a microplate reader Fluo Star Optima (Bmg Labtec GmbH, Ortenberg, Germany).

### Tumor growth inhibition in vivo

The SK-MES-1, H520, shCTL H520, shARG2 H520, and SW900 xenograft models were created by subcutaneous injection of 10^7^ corresponding cells in Matrigel in a 1:1 ratio (BD, Bio-science, San Jose, CA, USA) into the upper back of nude mice (female, 4-6-week-old, 10–14 g, BALB/cAnN-nu, Charles River Laboratories, Wilmington, USA). The sample size was estimated to be six, which was according to previous publication^23^, and all mice was included in analysis. Mice were randomized manually to one of the different groups after tumor size reached 50–100 mm^3^. Phosphate-buffered saline (as control) or BCT-100 (20 or 60 mg/kg, twice a week) was administered intraperitoneally. Tumor dimension (using standard calipers) and body weight of mice were measured twice a week and tumor volume calculated [volume = (length × width × height)/2]^24^. No blinding was done. For humane reasons, mice were sacrificed when tumor volume reached 600 mm^3^ or when significant tumor shrinkage was observed. Tumor xenografts and serum were collected for analysis. The study protocol was approved by the institutional Animal Ethics Committee (approval reference number: CULATR 3463-14), and standard humane endpoints for animal research were applied.

### Study of protein expression with Western blot

Western blot was performed as previously described^25^. Specific primary antibodies [mouse monoclonal anti-human β-actin (A1978) (Sigma-Aldrich, St. Louis, Missouri, United States), anti-ASS1 (SC-99178), anti-OTC (SC-102051), anti-Mcl-1 (SC-12756), anti-Bim (SC-374358), anti-CAT-1 (sc-515782) (Santa Cruz Biotechnology, Inc., Santa Cruz, CA, USA), anti-ARG1 (#93668), anti-ARG2 (#19324), anti-cyclin A2, B1, D3, E1 (#9869), anti-CDK4 (#9868), anti-Ki67 (#9449) (Cell Signaling Technology, Danvers, Massachusetts, USA), anti-PEG (31-1008-00) (RevMAb, San Francisco, USA) antibodies] and corresponding horseradish peroxidase (HRP)-conjugated secondary antibody (#7074 and #7076) (Cell Signaling Technology) were purchased. An enhanced chemiluminescence (ECL) kit (GE Healthcare) was used to detect protein expression. Beta-actin was selected as the house-keeping protein^22^. The quantification was measured using GelQuantNET software (Biochem Lab Solutions, CA, USA).

### Study of protein expression with immunohistochemistry

The ASS1, OTC, and ARG2 expression in human lung SCC patient samples was examined by immunohistochemical analysis of lung SCC tumors in paraffin-embedded sections. The research protocol was approved by the Institutional Review Board of the University of Hong Kong/Hospital Authority Hong Kong West Cluster (HKU/HA HKW IRB) (IRB reference number UW 18-019). Samples were collected and analyzed from 12 patients. No consent was required from all subjects. No patient photos were required. Immunohistochemistry staining was carried out according to the standard protocol. Photos were taken using a Nikon Ni-U fluorescence microscope (Nikon, Tokyo, Japan) equipped with a camera/detector Diagnostic Instrument RT3 Slider (Meyer Instruments, Houston, USA).

### Arginine concentration detection

An l-arginine ELISA kit was obtained (Immundiagnostik, Bensheim, Hessen, Germany) and the procedure conducted according to the manufacturer’s instructions. In brief, derivatized samples, control and standards were incubated with l-arginine antibody overnight at 4 °C. Peroxidase conjugate was added after washing. The reaction was terminated with stop solution after incubation with tetramethybenzidine substrate. Absorbance (450 nm) was measured with a reference (620 nm) using a microplate reader Fluo Star Optima.

### Terminal deoxynucleotidyl transferase-dUTP nick end labeling (TUNEL) assay

Click-iT^®^ Plus TUNEL Assay was purchased from Life Technologies. The procedures were carried out according to the manufacturer’s instructions. Formalin-fixed, paraffin-embedded tumor xenograft sections were obtained. First, deparaffinized, fixed and permeablized sections were incubated with terminal deoxynucleotidyl transferase (TdT) reaction buffer. Second, buffer was discarded and replaced by TdT buffer containing EdUTP, TdT, and TdT enzyme. Third, TUNEL reaction cocktail ((TUNEL reaction buffer, TUNEL reaction buffer additive), Alexa Fluor^®^ picoyl azide and copper protectant) was used to stain slides. Finally, slides were mounted with Prolong^®^ Gold antifade reagent containing DAPI. Photos were captured using a Nikon Ni-U fluorescence microscope.

### Statistical analysis

Experiments were repeated at least three times and data analyzed (mean ± standard error of the mean). The difference between groups (more than two groups) was analyzed using repeated measure or one-way analysis of variance by Prism (GraphPad Software, La Jolla, Southern California, USA). A *p* value < 0.05 determined statistical significance (^*^p < 0.05, ^**^p < 0.01, ^***^p < 0.001).
